# Clinical characteristics and genetic analysis of different Dandy-Walker malformation

**DOI:** 10.1186/s12884-026-09656-3

**Published:** 2026-07-13

**Authors:** Xingye Wei, Ping Huo, Xuedong Shi, Pingping Zhang, Ning Zhang, Yanmei Sun

**Affiliations:** Department of Reproductive Genetics, Hebei General Hospital, Shijiazhuang, 050051 P. R. China

**Keywords:** Dandy-Walker malformation, karyotype analysis, CNV-seq, SNP-array, WES

## Abstract

**Background:**

Dandy-Walker malformation (DWM) is a rare congenital malformation of the central nervous system (CNS). It is characterized by variable hypoplasia of the cerebellar vermis, cystic dilatation of the fourth ventricle, with or without an enlarged cisterna magna. Given that large-sample genetic analysis of DWM has not been conducted to date, this study aims to provide more comprehensive genetic counseling for prenatal diagnosis by summarizing the clinical features and genetic findings of DWM.

**Methods:**

Between January 2010 and May 2025, among 22,048 pregnant women, there were 487 cases of fetal DWM that underwent prenatal diagnosis. Karyotyping, copy number variation sequencing (CNV-seq), single-nucleotide polymorphism array (SNP-array) or whole-exome sequencing (WES) were employed for prenatal genetic diagnosis. The results of ultrasonography, genetic analyses, and prognoses were tabulated.

**Results:**

Among the 487 cases, 35 (7.19%) were diagnosed with classic Dandy-Walker malformation (cDWM), 59 (12.11%) with vermian hypoplasia (VH), and 393 (80.70%) with mega cisterna magna (MCM) according to ultrasonography. A total of 245 cases (50.31%) presented with additional ultrasound abnormalities; central nervous system (CNS) abnormalities were the most prevalent (21.36%), among which ventriculomegaly constituted the primary finding. Prenatal genetic abnormalities were detected in 50 cases (10.27%). Karyotyping identified chromosomal abnormalities in 42 cases (8.62%), including numerical abnormalities, structural abnormalities, and chromosomal mosaicism, with trisomy-18 (2.26%) being the most commonly detected. There were 8 cases with normal karyotypes, but among them, 6 cases were detected with chromosomal abnormalities by CNV-seq/SNP-array, and 2 cases were detected with gene mutations by WES. Advanced maternal age was associated with an increased risk of chromosomal abnormalities, a proportion of which may present with DWM. Among the 453 cases that underwent follow-up, the cDWM and VH groups exhibited a significantly higher incidence of pregnancy terminations compared to the MCM group. Subsequent follow-up of cases with continued pregnancies revealed 1 case of neonatal death in the VH group and 5 cases in the MCM group. Additionally, there was 1 case of abnormal postnatal development in the cDWM group and 3 cases in the MCM group.

**Conclusions:**

Pregnant women carrying DWM fetuses should undergo prenatal diagnosis including karyotyping, CNV-seq/SNP-array and WES if necessary, especially for fetuses with cDWM or VH, as the fetuses with cDWM or VH showed higher rates of genetic abnormalities. The pregnancy outcomes of most fetuses with MCM are favorable, while fetuses with cDWM or VH are prone to adverse gestational outcomes.

**Supplementary Information:**

The online version contains supplementary material available at https://doi.org/10.1186/s12884-026-09656-3.

## Background

Dandy-Walker malformation (DWM) is a rare congenital malformation of the CNS, characterized by varying degrees of cerebellar vermis hypoplasia, cystic dilatation of the fourth ventricle, with or without an enlarged cisterna magna. It belongs to the category of congenital posterior fossa malformations, with an incidence of approximately 1 in 25,000 to 35,000. DWM can be associated with CNS malformations or non-CNS malformations, and CNS malformations are more prevalent, followed by cardiovascular, urinary system, and digestive system malformations, etc [1–3]. Although DWM has been recognized for an extended period, its precise etiology and pathogenic mechanism remain unclear. Alcohol consumption, maternal diabetes, rubella and cytomegalovirus infections may contribute to the occurrence of DWM. A significant proportion of DWM patients exhibit genetic abnormalities, including chromosomal aneuploidies (e.g., trisomy 13, 18, and 21), chromosomal microdeletions (e.g., in regions 1q25.3-q32.1 and 3q22-q24), chromosome 9p duplication, and mutations in genes *ZIC1*, *ZIC4*, *FOXC1*, etc [1, 4, 5]. The diagnosis of fetal DWM relies primarily on ultrasound examination and/or magnetic resonance imaging (MRI). Advances in prenatal examination technology and increased awareness of prenatal screening have improved the detection rate of these abnormalities. Based on the degree of cerebellar vermis development, DWM is classified into classic Dandy-Walker malformation (cDWM), vermian hypoplasia (VH, also referred to as Dandy-Walker variant), and mega cisterna magna (MCM) [6]. In our previous study [1], we focused on cDWM and VH fetuses, detailing their clinical and genetic characteristics, but did not include MCM cases. To provide a more comprehensive understanding, this study incorporates MCM fetuses into the cohort to systematically describe the clinical features and genetic profiles of these three subtypes. To further elucidate the pathogenesis and prenatal diagnosis of DWM, the clinical data and genetic test results of 487 fetuses with DWM were summarized in this study.

## Methods

**Fig. 1:**
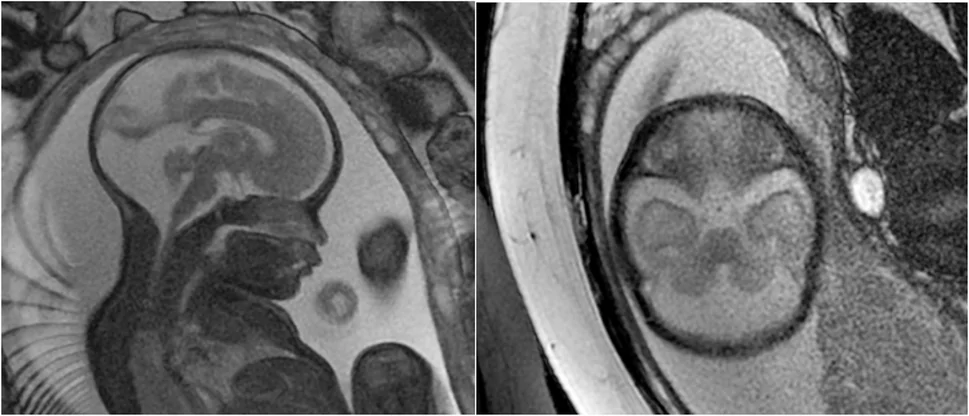
DWM, dysplasia of the cerebellar vermis, and enlarged cisterna magna communicates with the fourth ventricle

From January 2010 to May 2025, a total of 22,048 pregnant women underwent prenatal diagnosis, and 487 pregnant women whose fetuses presented with DWM underwent amniocentesis or cordocentesis under ultrasound guidance for genetic testing. Based on the degree of cerebellar vermis development, DWM is classified into cDWM, VH, and MCM. cDWM is a relatively rare condition characterized by the complete or partial absence of the cerebellar vermis, an enlarged cisterna magna that communicates with the dilated fourth ventricle; VH or Dandy-Walker variant is characterized by cerebellar vermian hypoplasia, cystic dilatation of the fourth ventricle, with a normal posterior fossa. MCM presents as a simple enlarged cisterna magna, without underdevelopment or dysplasia of cerebellar vermis, and with normal fourth ventricle and supratentorial structures of the cerebellum [6, 7]. MRI images showed DWM in the following Fig. 1.

The results of intracranial and extracranial ultrasound abnormalities, as well as genetic analysis results of the fetuses were tabulated. Fetal ultrasonography was conducted by experienced sonographers using a 5 MHz transducer (IU22, Philips; VOLUSON E8, GE). Karyotyping was performed for all 487 cases as the first-line prenatal diagnostic test following invasive sampling. CNV‑seq or SNP-array was performed in selected cases including fetuses with structural anomalies (one or multiple), sonographic soft markers highly associated with pathogenic copy number variations, or inconclusive karyotyping results, etc [8]. WES is indicated for fetuses with abnormal prenatal imaging findings (e.g., multiple congenital anomalies, skeletal abnormalities, cardiovascular malformations, central nervous system malformations) after conventional karyotyping and CNV testing have revealed no abnormalities, or for cases with suspected rare genetic syndromes, etc [9]. The choice of genetic testing was determined by the severity and complexity of sonographic findings, clinical indications, and parental preferences. All samples were subjected to rapid prenatal karyotyping, whereas only 104 and 5 cases underwent CNV-seq/SNP-array or WES, respectively. Statistical analyses were performed using the Statistical Package for the Social Sciences Version 27.0 (SPSS, IBM, Chicago, IL, USA). The chi-square test was employed for comparisons between groups (*P* = 0.05). To adjust for multiple comparisons, the Bonferroni method was applied, and the corrected significance threshold was set at 0.0167 (0.05/[3(3 − 1)/2]). Two-sided tests were performed, and *P* < 0.0167 were considered statistically significant. Multivariable logistic regression was performed to adjust for gestational age and sampling method, with a two-sided *P* < 0.05 considered significant.

## Results

Among 22,048 pregnant women, 487 cases with fetal DWM underwent invasive sampling procedures, including 65 amniocenteses and 422 cordocenteses. The median maternal ages were 28.00 (25.00,32.00) years, 28.00 (24.00,31.00) years, and 29.00 (25.50,32.00) years respectively. The maternal age data of the three groups did not follow a normal distribution (*P* < 0.05), and the differences were not statistically significant as evaluated by the Kruskal-Wallis H test (*P* > 0.05). The median gestational ages were 27.00 (20.00,31.00) weeks, 24.00 (20.00,29.00) weeks, and 31.70 (29.40,33.30) weeks respectively. The gestational age data of the three groups did not follow a normal distribution (*P* < 0.05), and the differences were statistically significant as evaluated by the Kruskal-Wallis H test (*P* < 0.05). The gestational weeks of the MCM group were significantly higher than those of the other two groups. (Table 1).

**Table 1:** Basic characteristics of 487 fetuses with DWM

	cDWM	VH	MCM
Age	28.00(25.00,32.00)	28.00(24.00,31.00)	29.00(25.50,32.00)
Gestational week	27.00(20.00,31.00)	24.00(20.00,29.00)	31.70(29.40,33.30)
Type of sample			
Amniotic fluid	13	35	17
Umbilical vein blood	22	24	376
Testing items			
Karyotype	21	40	322
Karyotype+CNV testing*	14	18	67
Karyotype+CNV-seq+ WES	0	1	4
Total	35	59	393

*CNV testing: CNV-seq or SNP-array

Among the 487 cases, 35 (7.19%) were diagnosed with cDWM, 59 (12.11%) with VH, and 393 (80.70%) with MCM. A total of 245 cases (50.31%) of DWM were accompanied by other ultrasound abnormalities. Among the other ultrasound-associated abnormalities of DWM, CNS malformations were the most frequently observed, accounting for 21.36%. The most common CNS malformation was ventriculomegaly, accounting for 14.37%. Cardiovascular system malformations accounted for 9.45%, and the most prevalent cardiovascular system abnormality was ventricular septal defect (3.70%). Other ultrasound-detected abnormalities included polyhydramnios (9.65%), urinary system abnormalities (9.03%), digestive system abnormalities (6.16%), single umbilical artery (2.87%), extremity abnormalities (2.26%), etc. (Table 2).

**Table 2:** Fetal DWM presenting with other ultrasound abnormalities

Ultrasound abnormalities	*n*	%^*^
Central Nervous System	104	21.36
Ventriculomegaly	70	14.37
Abnormal cavum septum pellucidum	18	3.70
Choroid plexus cyst	12	2.46
Reduced Transverse Cerebellar Diameter	2	0.41
Meningocele	1	0.21
Blake’s pouch cyst	1	0.21
Cardiovascular system	46	9.45
Ventricular septal defect	18	3.70
Persistent left superior vena cava	5	1.03
Complete endocardial cushion absence	4	0.82
Persistent right umbilical vein	4	0.82
Complex cardiovascular developmental malformations	4	0.82
Uneven heart development	3	0.62
Aberrant right subclavian artery	2	0.41
Absence of ductus venosus	2	0.41
Small internal diameter of the aortic arch	2	0.41
Ebstein anomaly	1	0.21
Aortic riding span	1	0.21
Urinary system	44	9.03
Widened renal pelvis	34	6.98
Enlarged or shrunken Kidney	5	1.03
Duplex kidney	2	0.41
Renal cyst	2	0.41
Horseshoe kidney	1	0.21
Digestive System	30	6.16
Gallbladder enlargement or undetected gallbladder	19	3.90
Small or large stomach vesicles	7	1.44
Digestive tract obstruction	4	0.82
Face and Neck	13	2.67
Nasal bone dysplasia	9	1.85
Cervical cystic hygroma	4	0.82
Extremity	11	2.26
Abnormal posture	5	1.03
Shortness	4	0.82
Polydactyly	2	0.41
Other		
Polyhydramnios	47	9.65
Single umbilical artery	14	2.87
Ascites or Pericardial effusion or Pleural effusion	12	2.46
Nuchal fold thickening	10	2.05
Nuchal translucency thickening	6	1.23
Visceral version	3	0.62
Umbilical cord cyst	2	0.41
Diaphragmatic hernia	2	0.41

Repeat counts if ≥ 2 ultrasound indices are abnormal in the same fetus

^*^Percentages are based on the total 487 fetuses

Among the 487 cases, 50 (10.27%) presented prenatal genetic abnormalities, including 13 (37.14%) in the cDWM group, 17 (28.81%) in the VH group, and 20 (5.09%) in the MCM group (Table 3). To account for the significant differences in gestational age across subgroups (Table 1), logistic regression was performed with adjustment for gestational age and sampling method. After adjustment, both the cDWM group (OR = 7.39, 95% *CI*:2.98–18.35, *P <* 0.001) and the VH group (OR = 4.48, 95% *CI*:1.81–11.06, *P* = 0.001) showed significantly higher genetic abnormality rates compared with the MCM group. No significant difference was observed between the cDWM and VH groups (OR = 1.65, 95% *CI*: 0.27–10.14, *P* = 0.287). Gestational age (*P* = 0.102) and sampling method (*P* = 0.855) were not statistically significant in the model, indicating that neither variable independently influenced the genetic abnormality rate after adjustment. To account for potential confounding by gestational age, a subgroup analysis restricted to cases diagnosed between 24 and 28 weeks of gestation after gestational age matching was performed; the results are provided in the supplementary materials (Table S1).

**Table 3:** Genetic abnormalities found in fetuses with DWM [n, (%)]

Groups	Total(*n*)	Abnormal results(*n*)	Frequency (%)
cDWM	35	13	37.14
VH	59	17	28.81
MCM	393	20	5.09

Including 8 cases with normal karyotype but abnormal CNV-seq, SNP-array, or WES

Among the 487 fetuses who underwent karyotype examination, 42 (8.62%) exhibited an abnormal karyotype. These included 5 cases (1.03%) of chromosomal mosaicism, 14 cases (2.87%) of chromosomal structural abnormalities, and 23 cases (4.72%) of chromosomal numerical abnormalities. The numerical abnormalities consisted of 11 cases of trisomy 18, 8 cases of trisomy 21, 3 cases of trisomy 13, and 1 case of triploidy (69, XXX), with trisomy 18 being the most commonly occurring. Structural abnormalities included chromosomal translocations, inversions, deletions, derivative chromosomes, marker chromosomes, etc. A more detailed list of abnormal karyotypes with chromosomal structures can be found in supplementary materials (Table S2). Among the 42 cases with chromosomal abnormalities, 12 originated from the cDWM group (*n* = 35, 34.29%), 16 were from the VH group (*n* = 59, 27.12%), and 14 were from the MCM group (*n* = 393, 3.56%), as shown in Table 4. Logistic regression was also performed with adjustment for gestational age and sampling method. After adjustment, both the cDWM group (OR = 9.22, 95% *CI*: 3.50–24.30, *P* < 0.001) and the VH group (OR = 5.98, 95% *CI*: 2.28–15.69, *P* < 0.001) showed significantly higher karyotype abnormality rates compared with the MCM group. No significant difference was observed between the cDWM and VH groups (OR = 0.648, 95% *CI*: 0.253–1.660, *P* = 0.366). Gestational age was independently associated with karyotype abnormalities (OR = 0.89, 95% *CI*: 0.80–0.99, *P* = 0.039), while sampling method was not significantly associated with karyotype abnormalities (*P* = 0.462).

**Table 4:** Results of chromosomal karyotyping analysis of fetuses with DWM[n, (%)]

Chromosome abnormality	Total [*n*^a^, (%)]	cDWM [*n*^b^, (%)]	VH [*n*^c^, (%)]	MCM [*n*^d^, (%)]
Numerical abnormalities	23(4.72)	7(20.00)	11(18.64)	5(1.27)
Trisomy−18	11(2.26)	2(5.71)	5(8.47)	4(1.02)
Trisomy−21	8(1.64)	3(8.57)	4(6.78)	1(0.25)
Trisomy−13	3(0.62)	1(2.86)	2(3.39)	0
69, XXX	1(0.21)	1(2.86)	0	0
Structural abnormalities	14(2.87)	5(14.29)	5(8.47)	4(1.02)
Chromosome mosaicism	5(1.03)	0	0	5(1.27)
46,XY/46,XX	1(0.21)	0	0	1(0.25)
47,XN,+9/46,XN	1(0.21)	0	0	1(0.25)
45,X/46,X, del(X)(p11)/46,X,+mar	1(0.21)	0	0	1(0.25)
45,X/46,X, i(X)(q10)/46,XX	1(0.21)	0	0	1(0.25)
45,X/46,XY	1(0.21)	0	0	1(0.25)
Total	42(8.62)	12(34.29)	16(27.12)	14(3.56)

^a^The percentage of cases in total 487 fetuses

^b^The percentage of cases in total 35 fetuses

^c^The percentage of cases in total 59 fetuses

^d^The percentage of cases in total 393 fetuses

CNV-seq/SNP-array was performed in 104 cases, and WES was performed in 5 cases, and the results demonstrated variable concordance with the karyotyping. Prenatal genetic abnormalities were also identified in 8 cases with normal karyotyping but abnormal CNV-seq/SNP-array or WES results. The copy number variants (CNVs) identified by SNP-array or CNV-seq included pathogenic CNVs, likey pathogenic CNVs and variants of uncertain significance(VUS). CNV-seq/SNP-array identified 6 chromosomal aberrations, including 2 microdeletions, 3 microduplications, and 1 case of chromosome 12 mosaicism with a 20% mosaicism level. WES identified pathogenic or likely pathogenic gene mutations in 2 cases, involving *RAF1*, *KDM6A*, and *GAA* gene.

Among the 487 pregnant women, 64 (13.14%) were of advanced maternal age and 16 (25%) of these women had fetal chromosomal abnormalities; 423 (86.86%) were of non-advanced maternal age and 32 (7.57%) of these women had fetal chromosomal abnormalities. Meanwhile, we further stratified the analysis by maternal age within each of the three subgroups. The rate of fetal chromosomal abnormalities was consistently higher in the advanced maternal age group compared to the younger maternal age group (all *P* < 0.05).(Table 5).

**Table 5:** Association of maternal age with chromosomal abnormalities

Group	Maternal age	Total/*n*	Chromosomal abnormalities [*n*, (%)]
cDWM	< 35 years	31	9(29.03%)
	≥ 35 years	4	4(100%)
VH	< 35 years	51	10(19.61%)
	≥ 35 years	8	6(75.00%)
MCM	< 35 years	341	13(3.81%)
	≥ 35 years	52	6(11.54%)

Including 6 cases with normal karyotype but abnormal SNP-array, CNV-seq

As presented in Table 6, a follow-up was conducted on 453 cases of fetal DWM. A total of 99 (21.85%) pregnancies opted for pregnancy termination, among which 24 (70.59%) cases were in the cDWM group, 42 (76.36%) in the VH group, and 33 (9.07%) in the MCM group. The incidence of pregnancy terminations in the cDWM or VH group was significantly higher than that in the MCM group (*P* < 0.001 for both comparisons after Bonferroni correction). A total of 354 (78.15%) cases continued the pregnancy.

Nevertheless, 1 case of neonatal death was noted in the VH group, whereas 5 cases were documented in the MCM group. One of these cases was associated with cytomegalovirus infection, which could be the cause of death in this case. Additionally, prenatal genetic examinations conducted on these six fetuses revealed no genetic abnormalities. Among the live-birth cases, there was one newborn with developmental abnormalities in the cDWM group and three newborns in the MCM group. These cases manifested as severe sensorineural deafness, somnolence, lethargy, feeding difficulties, swallowing difficulties, epilepsy, or hypertonia. In these four cases, pathogenic chromosomal microdeletions or gene mutations were detected in two cases via CNV-seq or WES. Detailed information on cases of neonatal death and abnormal postnatal development can be found in Table 7.

**Table 6:** Pregnancy outcomes of fetuses with DWM

Group	Total (*n*)	follow-up(n)	Induced abortion[n, (%)]	neonatal death(n)	Normal postnatal development(n)
cDWM	35	34	24(70.59%)*	0	9
VH	59	55	42(76.36%)*	1	12
MCM	393	364	33(9.07%)	5	323

*P < 0.001 for comparisons after Bonferroni correction (cDWM vs. MCM; VH vs. MCM)

**Table 7:** Fetal DWM with neonatal death and abnormal postnatal development

Age	Ultrasound Findings	Genetic testing	Follow-up
Case 1	VH Ventriculomegaly	Karyotyping: normal SNP-array: normal	A female infant born at full term and died 7 days after birth with multiple system malformations
Case 2	MCM	Karyotyping: normal	A female infant born at full term and died 20 days after birth with cleft palate and feeding difficulties
Case 3	MCM Pleural effusion	Karyotyping: normal	A male infant born at full term and died prematurely with pleural effusion
Case 4	MCM Ventricular septal defect Widened renal pelvis Single umbilical artery	Karyotyping: normal CNV-seq: VUS	A male infant was delivered at 35 weeks’gestation due to fetal distress and died 3 months after birth with intracranial hemorrhage and ventricular septal defect
Case 5	MCM Pleural effusion	Karyotyping: normal CNV-seq: normal WES: normal	A male infant was delivered at 33 weeks’gestation due to fetal distress and no vital signs at birth, presented with edema
Case 6	MCM Ventriculomegaly	Karyotyping: normal CNV-seq: VUS and cytomegalovirus infection WES: normal	A male infant born at full term and died 3 days after birth with hypertonia, hepatosplenomegaly, and thrombocytopenia
Case 7	MCM Polyhydramnios Single umbilical artery	Karyotyping: normal CNV-seq: normal WES: pathogenic mutation of *RAF1*	A male infant was delivered at 32^+ 4^ weeks’gestation due to premature rupture of membranes and presented with poor swallowing function
Case 8	MCM	Karyotyping: normal CNV-seq: normal	A female infant born at full term and presented with epilepsy, hypertonia
Case 9	MCM Enlarged gallbladder	Karyotyping: normal CNV-seq: pathogenic CNVs	A male infant born at full term and presented with somnolence, lethargy, feeding difficulties
Case 10	cDWM Ventriculomegaly	Karyotyping: normal	A female infant born at full term and presented with severe sensorineural deafness

## Discussion

Fetal ultrasound and MRI are efficacious approaches for detecting DWM. During prenatal screening, DWM is frequently associated with other CNS malformations or non-CNS malformations, including ventriculomegaly, corpus callosum dysplasia, meningocele, congenital cardiovascular malformations, renal system malformations, limb abnormalities, etc. The other ultrasound anomalies were predominantly observed among CNS malformations, with ventriculomegaly being the most prevalent. Subsequently, cardiovascular system malformations and renal system abnormalities were also commonly detected [1, 5]. This study also yielded consistent results, and polyhydramnios was also frequently observed among the accompanying abnormalities. Further in-depth investigation into the relationship between these systems’ abnormalities and DWM can provide more references for early screening and intervention. Therefore, a comprehensive evaluation of these systems during prenatal examinations is essential to improve the early detection of DWM, especially in cases involving the CNS, cardiovascular system, or polyhydramnios.

Genetic factors play an important role in the etiology of DWM. In the present study, the abnormality rate of prenatal genetic analysis was 10.27%, with the rates in the three groups being 37.14% (*n* = 13), 28.81% (*n* = 17), and 5.09% (*n* = 20), respectively. The abnormality rates in both the cDWM group and the VH group were significantly higher than that in the MCM group. This finding implies that the incidence of genetic abnormalities in cases featuring a simple enlarged cisterna magna without cerebellar vermis dysplasia is notably lower. When fetal cerebellar vermis dysplasia is present, the risk of genetic abnormalities increases, particularly chromosomal aneuploidy, which is consistent with the results of other studies [10–12]. Although the abnormal rate in the cDWM group was higher than that in the VH group, statistical significance was not observed. Given that not all cases in this study underwent karyotype analysis, CNV-seq/SNP-array and WES, the actual rate of genetic abnormalities is likely higher than the rate reported herein. Furthermore, advanced maternal age is associated with an increased risk of chromosomal abnormalities, a proportion of which may present with DWM.

The genetic abnormalities identified in this study included aneuploidy, chromosomal structural abnormalities, pathogenic/likely pathogenic CNVs, chromosomal mosaicism, and gene mutations, which were consistent with previous research [1, 4, 13]. Trisomy 18 was the most prevalent chromosomal abnormality in this study. Karyotype analysis did not always exhibit consistency with CNV-seq/SNP-array and WES, particularly in the detection of chromosomal structural abnormalities and gene mutations, where discrepancies were observed. Karyotype analysis is only capable of detecting chromosomal fragments larger than 5 Mb and is ineffective against submicroscopic chromosomal aberrations. CNV-seq/SNP-array can compensate for this deficiency and detect pathogenic/likely pathogenic CNVs [14–17].

Previous research has indicated that DWM fetuses may be linked to mutations in genes such as *ZIC2*, *ZIC4*, *FOXC1*, *SIL1*, and *EXOC3L2* [4, 13]. In the present study, WES identified pathogenic or likely pathogenic mutations in 2 cases, involving the *KDM6A*, *GAA*, *RAF1* gene and no chromosomal aberrations were found. WES has emerged as a potent tool for prenatal diagnosis in cases with abnormal ultrasound findings or congenital malformations, playing a significant role in identifying pathogenic or likely pathogenic mutations associated with clinical phenotypes. WES has improved the genetic diagnostic rates of fetuses with ultrasound abnormalities, particularly in cases involving kidney abnormalities, amniotic fluid abnormalities, and nervous system anomalies, etc [18, 19]. Therefore, karyotyping, CNV-seq/SNP-array, and WES are recommended for fetuses with DWM.

Maternal primary cytomegalovirus (CMV) infection exerts a significant impact on the development and prognosis of the fetus, with manifestations including ventriculomegaly, fetal growth restriction, and enlarged cisterna magna, etc. Congenital infection may result in sensorineural hearing loss and central nervous system involvement [20–22]. Therefore, it is crucial to conduct comprehensive CMV testing during early pregnancy.

The clinical manifestations of DWM include mental and motor developmental delay, hydrocephalus, increased intracranial pressure, spastic paralysis, and hearing impairment [5, 23]. The prognosis of DWM is associated with multiple factors, such as the presence of chromosomal abnormalities or genetic mutations, other ultrasound abnormalities, the degree of cerebellar vermis absence, and the severity of hydrocephalus [5]. It is extremely challenging to accurately assess the prognosis solely based on ultrasound findings. Even if no abnormalities are detected in the fetal genetic testing, some families may still choose to terminate the pregnancy due to concerns about potential adverse outcomes [1, 10, 12], especially in the cDWM group and the VH group. Although most fetuses with isolated MCM have favorable outcomes, adverse postnatal events (including neonatal death and neurodevelopmental abnormalities) can still occur in a small proportion of cases, sometimes due to non-genetic causes such as congenital cytomegalovirus infection. Therefore, postnatal follow-up remains necessary even when prenatal genetic testing is negative.

This study further analyzed the genetic and clinical characteristics of DWM to enhance the comprehension of this condition. When an enlarged cisterna magna is detected via ultrasound, it is imperative to conduct a further examination of the integrity of the cerebellar vermis. Fetal MRI can serve as a complementary tool to the ultrasound examination for achieving a definitive diagnosis. Transvaginal neurosonography represents a targeted high-resolution ultrasound technique that substantially improves the assessment of the fetal posterior fossa, particularly the cerebellar vermis, enabling detailed qualitative and quantitative evaluation of this structure. When used in addition to standard ultrasound and fetal MRI, transvaginal neurosonography serves as a valuable complementary tool, offering high-resolution anatomical detail that optimizes prenatal evaluation of posterior fossa anomalies [24, 25]. Upon the identification of DWM, genetic analysis should be carried out on the affected fetuses. Karyotype analysis in combination with CNV-seq/SNP-array can screen out the majority of chromosomal aberrations, and WES can detect potential pathogenic gene mutations. In this study, several gene mutations potentially associated with DWM were detected, which broadens the genetic spectrum of this disorder.

Several limitations of this study should be acknowledged. This is a retrospective study, the definition of ‘genetic abnormality’ in this study was a composite outcome encompassing karyotypic abnormalities, CNVs, and gene mutations. However, the coverage of testing methods varies across subgroups, all 487 cases underwent karyotyping, but only 104 cases were tested by CNV-seq/SNP-array, and only 5 by WES. Consequently, the reported rates of genetic abnormalities in the DWM fetuses may underestimate the true prevalence, particularly for submicroscopic CNV abnormalities and gene mutations that are not detectable by karyotyping alone. Future multicenter studies with larger sample sizes are warranted to systematically collect cases that undergo karyotyping, CNV testing, and WES, in order to more accurately estimate the true genetic abnormality rates in DWM fetuses. Additionally, subtype classification relies solely on prenatal ultrasound, lacking MRI validation or diagnostic consistency assessment. While ultrasound is the primary screening modality for fetal posterior fossa anomalies, it has inherent limitations in assessing cerebellar vermis development and fourth ventricular morphology, particularly when distinguishing mild vermis hypoplasia from MCM. Fetal MRI can serve as an essential auxiliary tool to ultrasound for achieving a more definitive diagnosis and refining subtype classification. The absence of MRI may have introduced classification bias in this study. Future studies should incorporate systematic MRI evaluation and ultrasound-MRI concordance analysis to improve diagnostic accuracy.

## Conclusion

Genetic factors contribute to a subset of fetal DWM cases, particularly cDWM and VH. Fetal DWM are frequently accompanied by other ultrasound abnormalities, with the CNS abnormalities are the most prevalent. Fetal isolated MCM carries a low risk of chromosomal abnormalities and is generally associated with favorable outcomes; however, adverse postnatal events can still occur in a small proportion of cases, sometimes due to non-genetic causes. Therefore, postnatal follow-up remains necessary even when prenatal genetic testing is negative. However, for the cDWM or VH, the incidence of chromosomal aberrations increases significantly and tending towards a poor prognosis. Consequently, prenatal detection of DWM need detailed evaluation of fetal systemic structural abnormalities, with particular emphasis on the central nervous and cardiovascular systems. It is necessary to carry out further prenatal genetic testing including karyotype analysis, copy number variation sequencing and WES.

## Supplementary Information

Supplementary Material 1.

## Data Availability

The variation data from the Whole-Exome Sequencing analysis reported in this study have been deposited in the Genome Variation Map (GVM) [26] in National Genomics Data Center, Beijing Institute of Genomics, Chinese Academy of Sciences and China National Center for Bioinformation [27], under accession number GVM001333. The data deposited in GVM are currently in a confidential state to protect patient privacy. Access to the data is available from the corresponding author upon reasonable request.

## Abbreviations

DWM: Dandy - Walker malformation
cDWM: classic Dandy - Walker malformation
MCM: Mega cisterna magna
CNS: Central nervous system
CNV-seq: Copy number variation sequencing
CNV: Copy number variation
SNP: Single-nucleotide polymorphism
VH: Vermian hypoplasia
WES: Whole-exome sequencing

## References

[1] Sun Y, Wang T, Zhang N, Zhang P, Li Y. Clinical features and genetic analysis of Dandy-Walker syndrome. BMC Pregnancy Childbirth. 2023;23(1):40. 10.1186/s12884-023-05367-1 PMC9847126 36653756

[2] D’Antonio F, Khalil A, Garel C, Pilu G, Rizzo G, Lerman-Sagie T, et al. Systematic review and meta-analysis of isolated posterior fossa malformations on prenatal ultrasound imaging (part 1): nomenclature, diagnostic accuracy and associated anomalies. Ultrasound Obstet Gynecol. 2016;47(6):690–7. 10.1002/uog.14900 25970099

[3] Wüest A, Surbek D, Wiest R, Weisstanner C, Bonel H, Steinlin M, et al. Enlarged posterior fossa on prenatal imaging: differential diagnosis, associated anomalies and postnatal outcome. Acta Obstet Gynecol Scand. 2017;96(7):837–43. 10.1111/aogs.13131 28295149

[4] Shalata A, Lauhasurayotin S, Leibovitz Z, Li H, Hebert D, Dhanraj S, et al. Biallelic mutations in EXOC3L2 cause a novel syndrome that affects the brain, kidney and blood. J Med Genet. 2019;56(5):340–6. 10.1136/jmedgenet-2018-105421 30327448

[5] Monteagudo A. Dandy-Walker Malformation. Am J Obstet Gynecol. 2020;223(6):B38–41. 10.1016/j.ajog.2020.08.184 33168220

[6] Karsonovich T, Das JM, Ahmad T. Dandy-Walker Malformation. StatPearls. Treasure Island (FL): StatPearls Publishing Copyright © 2026. StatPearls Publishing LLC.; 2026. 30855785

[7] Zhang XB, Gu YQ, Sun XF, Wang YN, Wang AC. [Dandy-Walker complex: a clinicopathologic study of 9 cases]. Zhonghua Bing Li Xue Za Zhi. 2013;42(12):815–8. 24507099

[8] Committee For Birth Defect P, Control Chinese Association Of Preventive Medicine, Genetic T, Precision Medicine Branch Chinese Association Of, Birth H, Hu T, Liu S. [Guidelines for the application of copy number variation testing in prenatal diagnosis]. Zhonghua Yi Xue Yi Chuan Xue Za Zhi. 2020;37(9):909–17. 32820498

[9] Application Collaboration Group Of Whole Exome Sequencing In Prenatal Diagnosis, Lou G, Hou Q, Yang K, Guo L. [Expert consensus on the application of prenatal exome sequencing for fetal structural anomalies]. Zhonghua Yi Xue Yi Chuan Xue Za Zhi. 2022;39(5):457 – 63. 10.3760/cma.j.cn511374-20210920-00765 35598257

[10] Forzano F, Mansour S, Ierullo A, Homfray T, Thilaganathan B. Posterior fossa malformation in fetuses: A report of 56 further cases and a review of the literature. Prenat Diagn. 2007;27(6):495–501. 10.1002/pd.1722 17367101

[11] Şeker E, Aslan B, Aydın E, Koç A. Long-term outcomes of fetal posterior fossa abnormalities diagnosed with fetal magnetic resonance imaging. J turk-ger gynecol a. 2023;24(1):28–32. 10.4274/jtgga.galenos.2022.2022-7-13 PMC10019011 36583295

[12] D’Antonio F, Khalil A, Garel C, Pilu G, Rizzo G, Lerman-Sagie T, et al. Systematic review and meta-analysis of isolated posterior fossa malformations on prenatal imaging (part 2): neurodevelopmental outcome. Ultrasound Obstet Gynecol. 2016;48(1):28–37. 10.1002/uog.15755 26394557

[13] Luo Y, Sun Y, Qian Y, Shen M, Wang L, Jin F, et al. [Genetic diagnosis of a fetus with Dandy-Walker syndrome]. Zhonghua Yi Xue Yi Chuan Xue Za Zhi. 2020;37(1):8–11. 10.3760/cma.j.issn.1003-9406.2020.01.003 31922586

[14] Lei T, Feng JL, Xie YJ, Xie HN, Zheng J, Lin MF. Chromosomal aneuploidies and copy number variations in posterior fossa abnormalities diagnosed by prenatal ultrasonography. Prenat Diagn. 2017;37(11):1160–8. 10.1002/pd.5159 28940600

[15] Sun Y, Zhang N, Tian H, Zhang P, Li Y. Prenatal diagnosis of the Dandy-Walker malformation associated with partial trisomy 12p and distal 15q deletion. J Genet. 2021;100:40. 34238780

[16] Zhi Y, Liu L, Cui S, Li Y, Chen X, Che J, et al. Pathogenic/likely pathogenic copy number variations and regions of homozygosity in fetal central nervous system malformations. Arch Gynecol Obstet. 2022;308(6):1723–35. 10.1007/s00404-022-06866-w 36464758

[17] Huang Y, Fu S, Shao D, Yao Y, Wu F, Yao M. Comprehensive chromosomal abnormality detection: integrating CNV-Seq with traditional karyotyping in prenatal diagnostics. BMC Med Genomics. 2025;18(1):81. 10.1186/s12920-025-02139-y PMC12042461 40301840

[18] Shboul M, Badaro N, Darweesh R, Fathallah R, El-Khateeb M, Al-Kuran O. Whole-exome sequencing for prenatal diagnosis of fetal anomalies: insights from a Jordanian cohort. J Matern Fetal Neonatal Med. 2025;38(1):2538208. 10.1080/14767058.2025.2538208 40721351

[19] Traversa A, Bernardo S, Paiardini A, Giovannetti A, Marchionni E, Genovesi ML, et al. Prenatal whole exome sequencing detects a new homozygous fukutin (FKTN) mutation in a fetus with an ultrasound suspicion of familial Dandy-Walker malformation. Mol Genet Genomic Med. 2020;8(1):e1054. 10.1002/mgg3.1054 PMC6978243 31756055

[20] Kang H, Wang L, Chen Y, Dong T, Pan Y, Liu Q. Screening and prenatal diagnosis of fetal cytomegalovirus infection: experience in a western Chinese city. BMC Infect Dis. 2025;25(1):542. 10.1186/s12879-025-10910-w PMC12004672 40240971

[21] Zhang L, Lv W, Ya J, Li Y, Yuan D, Cai P, et al. Clinical characteristics, pregnancy outcomes, and neonatal effects of cytomegalovirus infection in pregnant women in Liuzhou city, North central Guangxi of Southern China: a retrospective study from 2018 to 2024. BMC Pregnancy Childbirth. 2025;25(1):909. 10.1186/s12884-025-08083-0 PMC12400692 40890649

[22] Lipitz S, Elkan Miller T, Yinon Y, Weissbach T, De-Castro H, Hoffman C, et al. Revisiting short- and long-term outcome after fetal first-trimester primary cytomegalovirus infection in relation to prenatal imaging findings. Ultrasound Obstet Gynecol. 2020;56(4):572–8. 10.1002/uog.21946 31858642

[23] Stambolliu E, Ioakeim-Ioannidou M, Kontokostas K, Dakoutrou M, Kousoulis AA. The Most Common Comorbidities in Dandy-Walker Syndrome Patients: A Systematic Review of Case Reports. J Child Neurol. 2017;32(10):886–902. 10.1177/0883073817712589 28635420

[24] Malinger G, Ginath S, Lerman-Sagie T, Watemberg N, Lev D, Glezerman M. The fetal cerebellar vermis: normal development as shown by transvaginal ultrasound. Prenat Diagn. 2001;21(8):687–92. 10.1002/pd.137 11536272

[25] Paladini D, Malinger G, Birnbaum R, Monteagudo A, Pilu G, Salomon LJ, et al. ISUOG Practice Guidelines (updated): sonographic examination of the fetal central nervous system. Part 2: performance of targeted neurosonography. Ultrasound Obstet Gynecol. 2021;57(4):661–71. 10.1002/uog.23616 33734522

[26] Genome Variation Map. a worldwide collection of genome variations across multiple species. Nucleic Acids Res. 2021;49(D1):D1186-D1191. PMID = 33170268. 10.1093/nar/gkaa1005 PMC7778933 33170268

[27] CNCB-NGDC Members and Partners. Database Resources of the National Genomics Data Center, China National Center for Bioinformation in 2024. Nucleic Acids Res. 2024;52(D1):D18-D32. PMID = 38018256. 10.1093/nar/gkad1078 PMC10767964 38018256

