# Supplementary material for: Clinical characteristics and genetic analysis of different Dandy-Walker malformation
Source: BMC Pregnancy Childbirth. 2026 Jul 13;26:1118. doi: 10.1186/s12884-026-09656-3 (PMC13644209; doi:10.1186/s12884-026-09656-3)
Supplement: Supplementary file 1 [file 12884_2026_9656_MOESM1_ESM.doc]

Table S1. Genetic abnormalities found in fetuses with DWM at 24-28 weeks of gestation

| **Groups** | **Total(n)** | **Abnormal results(n)** | **Frequency (%)** |
| --- | --- | --- | --- |
| cDWM | 9 | 4 | 44.44 |
| VH | 16 | 6 | 37.50* |
| MCM | 64 | 6 | 9.38 |

**P*=0.012 < 0.0167, VH vs. MCM (Bonferroni-corrected threshold)

Table S2. Detailed karyotypes of structural chromosomal abnormalities (n=14)

| **Chromosome abnormality** | Total  [n^a^, (%)] | cDWM  [n^b^, (%)] | VH  [n^c^, (%)] | MCM  [n^d^, (%)] |
| --- | --- | --- | --- | --- |
| **Structural abnormalities** | 14(2.87) | 5(14.29) | 5(8.47) | 4(1.02) |
| 47,XN,del(1)(q42) | 1(0.21) | 0 | 1(1.69) | 0 |
| 46,XN,add(20)(p13) | 1(0.21) | 0 | 1(1.69) | 0 |
| 47,XN,+Mar | 1(0.21) | 0 | 1(1.69) | 0 |
| 46,XN,t(10;21)(p10;q10) | 1(0.21) | 0 | 1(1.69) | 0 |
| 47,XN,+der(14)t(8;14)(p11;q11) | 1(0.21) | 0 | 1(1.69) | 0 |
| 46,XN,inv(9)(p11q13) | 1(0.21) | 0 | 0 | 1(0.25) |
| 46,XN,t(13;21)(q12;q22) | 1(0.21) | 0 | 0 | 1(0.25) |
| 46,X,inv(Y)(p11q11) | 1(0.21) | 0 | 0 | 1(0.25) |
| 46,XN,t(3;9)(p13;q22) | 1(0.21) | 0 | 0 | 1(0.25) |
| 46,XN,add(8)(p11) | 1(0.21) | 1(2.86) | 0 | 0 |
| 46,XN,der(2)t(2;11)(q35;q13) | 1(0.21) | 1(2.86) | 0 | 0 |
| 46,XN,rec(3)dup(3q)inv(3)(p25q23) | 1(0.21) | 1(2.86) | 0 | 0 |
| 46,XN,der(8)(8pter-8q24::12p10-12qter),i(12)(p10) | 1(0.21) | 1(2.86) | 0 | 0 |
| 46,XN,t(7;8)(p21;q43) | 1(0.21) | 1(2.86) | 0 | 0 |

^a^ The percentage of cases in total 487 fetuses

^b^ The percentage of cases in total 35 fetuses

^c^ The percentage of cases in total 59 fetuses

^d^The percentage of cases in total 393 fetuses
